# Supplementary material for: Sulfatase 2 Affects Polarization of M2 Macrophages through the IL-8/JAK2/STAT3 Pathway in Bladder Cancer
Source: Cancers (Basel). 2022 Dec 26;15(1):131. doi: 10.3390/cancers15010131 (PMC9818157; doi:10.3390/cancers15010131)

Figure 2B

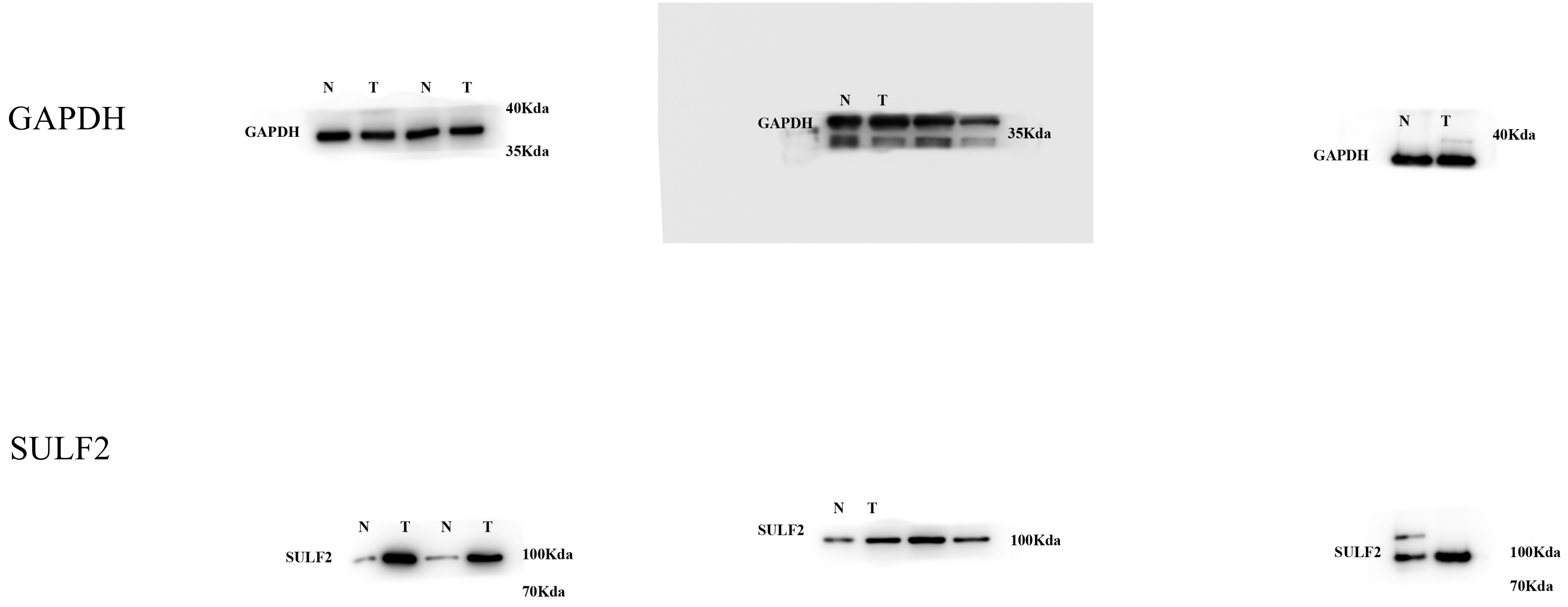
GAPDH
N T N T
40Kda
GAPDH
35Kda
N T
GAPDH
35Kda
N T
40Kda
GAPDH
SULF2
N T N T
SULF2
100Kda
70Kda
N T
SULF2
100Kda
SULF2
100Kda
70Kda

Figure2D

GAPDH

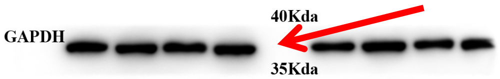


SULF2

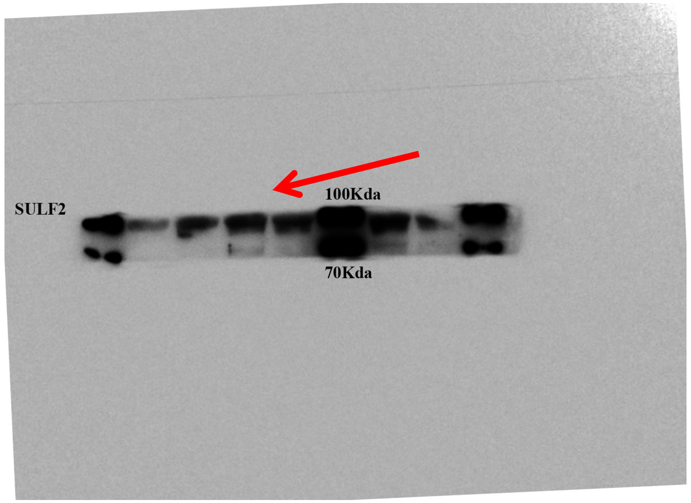

Figure 2E

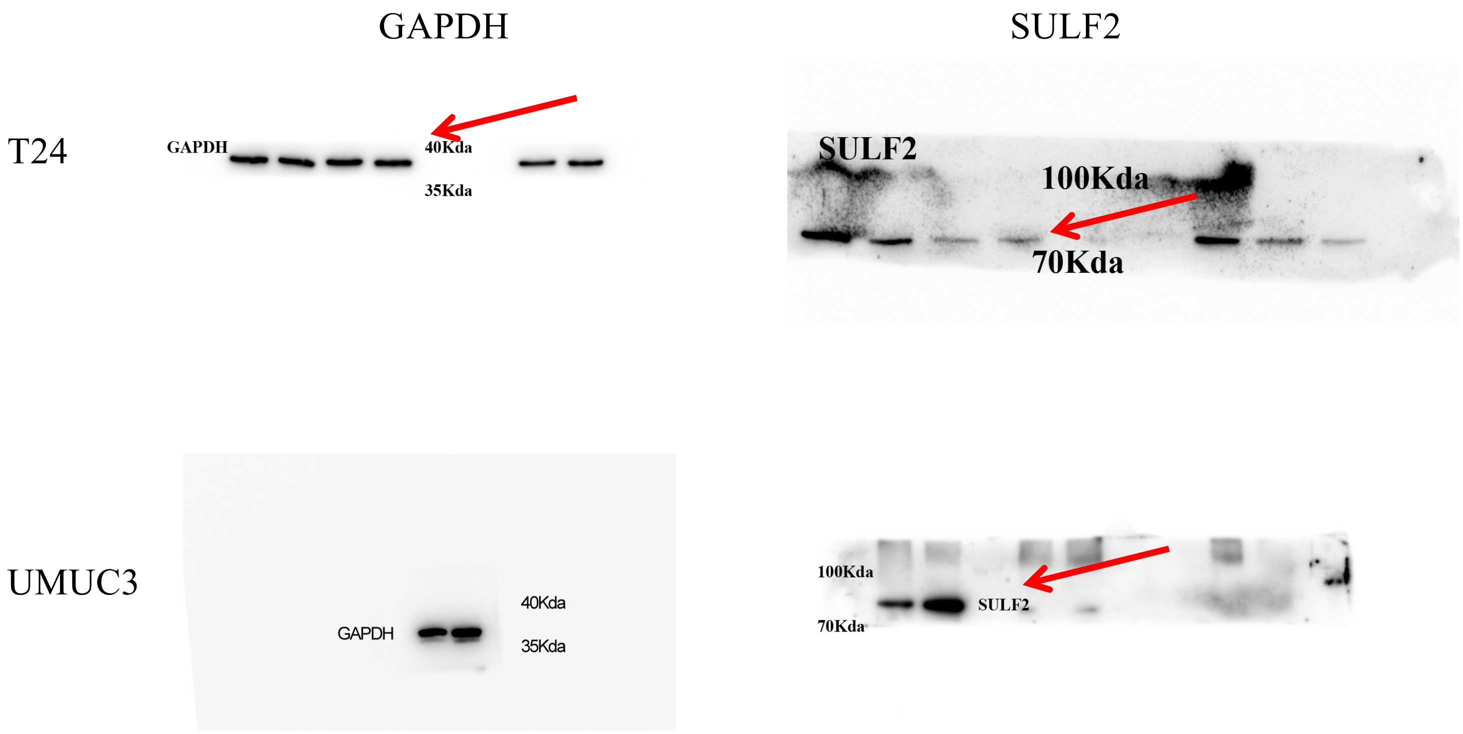
GAPDH
SULF2
T24
GAPDH
40Kda
35Kda
SULF2
100Kda
70Kda
UMUC3
GAPDH
40Kda
35Kda
100Kda
SULF2
70Kda

Figure4D

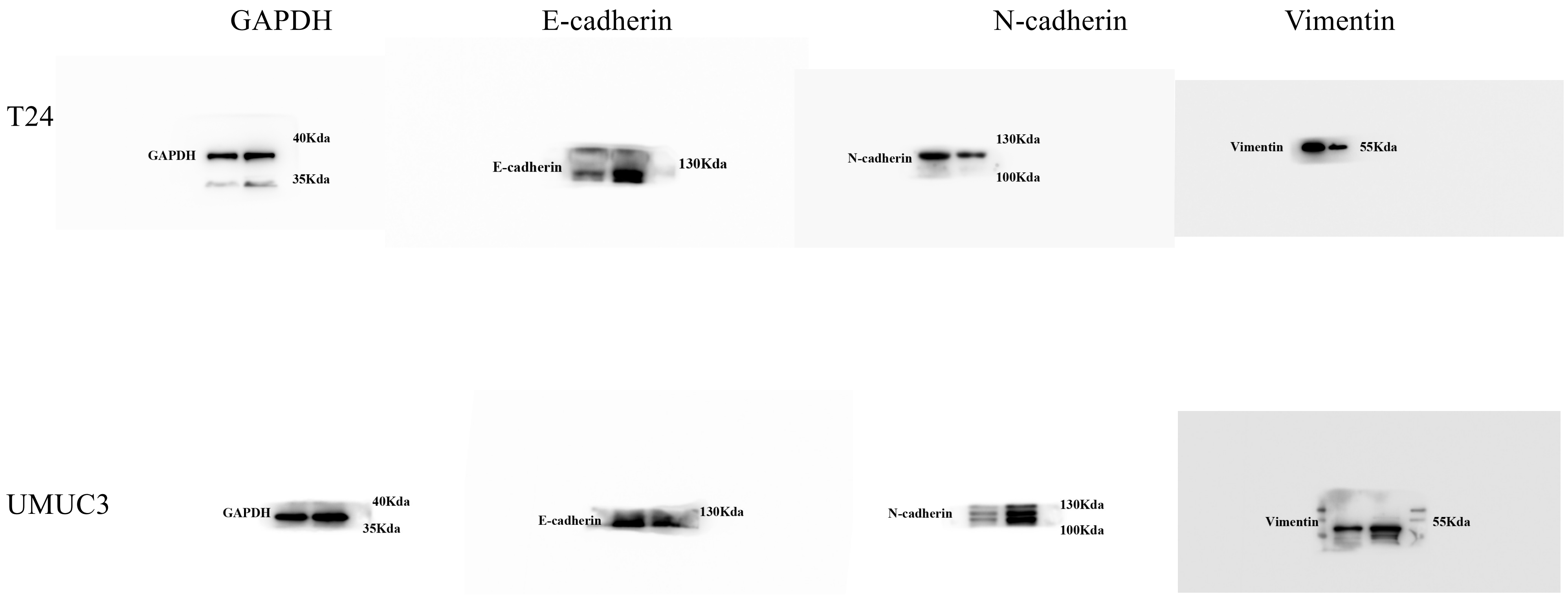
GAPDH
E-cadherin
N-cadherin
Vimentin
T24
GAPDH
40Kda
35Kda
E-cadherin
130Kda
N-cadherin
130Kda
100Kda
Vimentin
55Kda
UMUC3
GAPDH
40Kda
35Kda
E-cadherin
130Kda
N-cadherin
130Kda
100Kda
Vimentin
55Kda

Figure6B

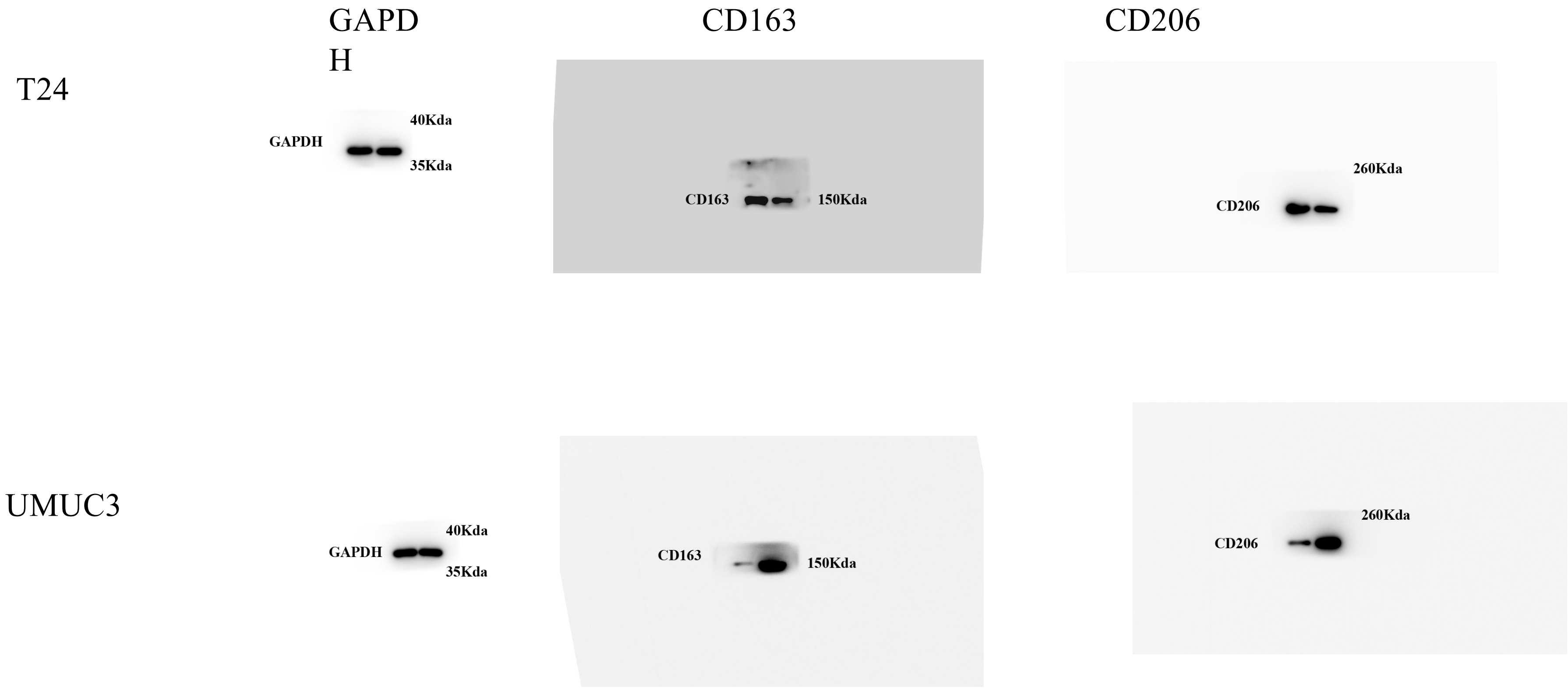
GAPDH
CD163
CD206
T24
GAPDH
40Kda
35Kda
CD163
150Kda
CD206
260Kda
UMUC3
GAPDH
40Kda
35Kda
CD163
150Kda
CD206
260Kda

Figure6E

T24

GAPD
H

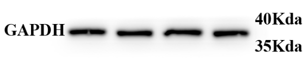


CD163

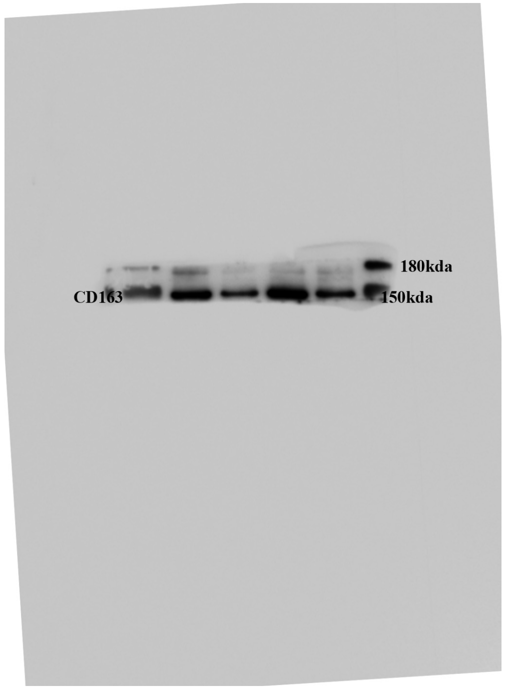


CD206

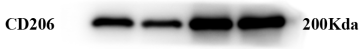

Figure6F

GAPD
H

CD163

CD206

UMUC3

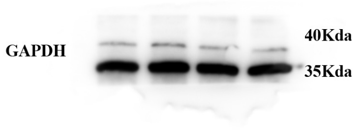


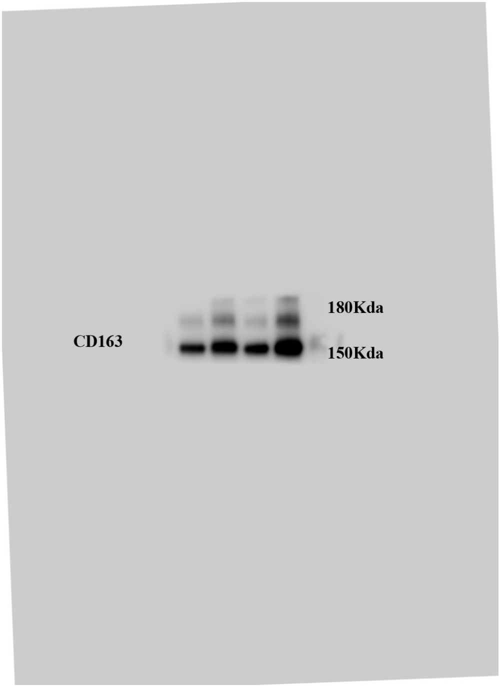


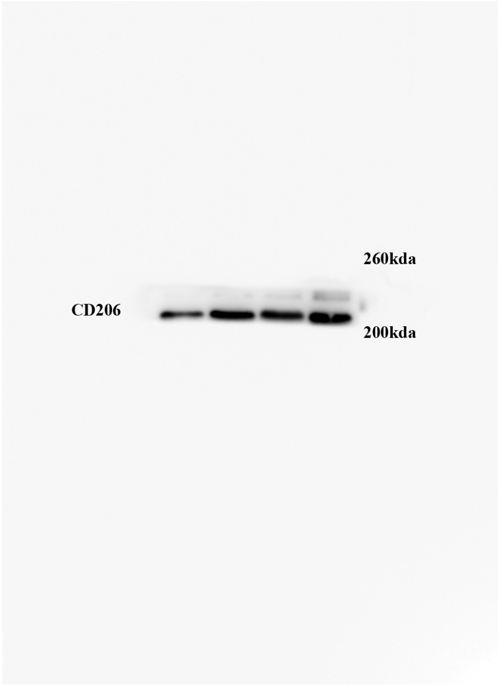

Figure6H

GAPD
H

CD163

CD206

THP1

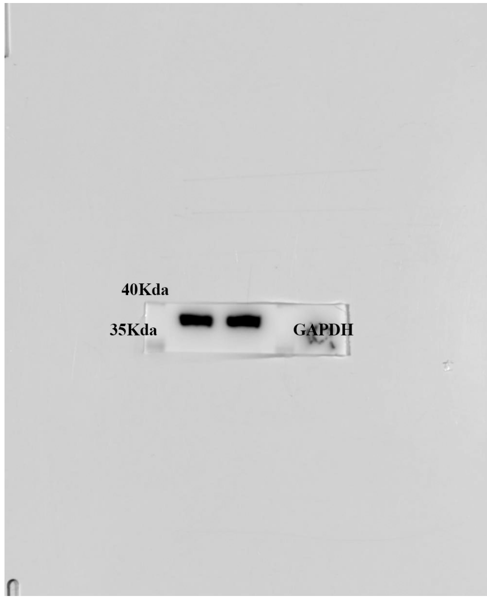


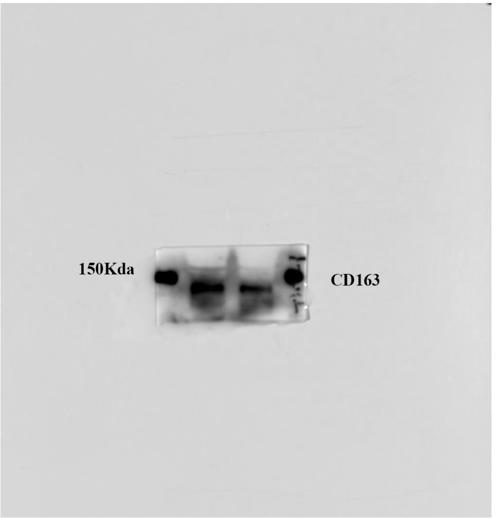


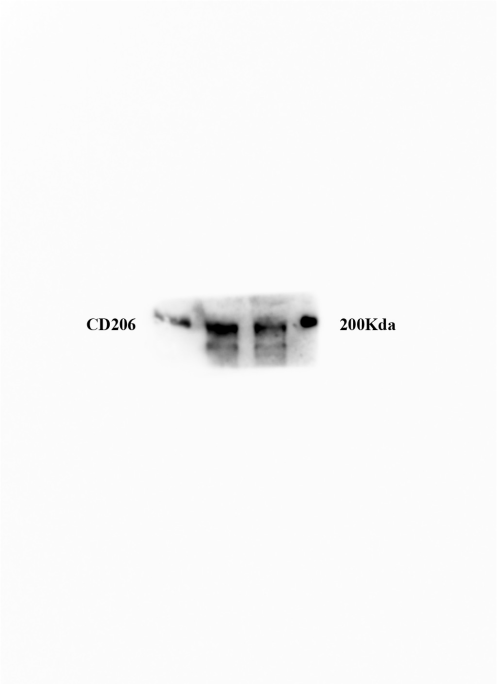

Figure7B

p-JAK2

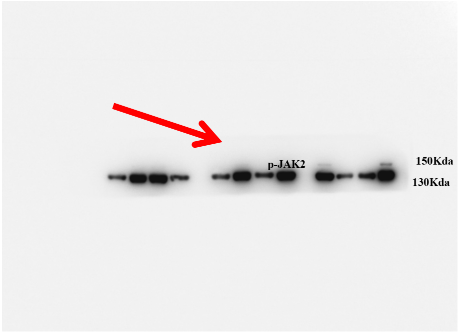


JAK2

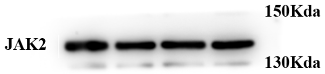


STAT3

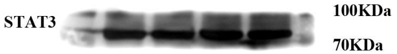


p-STAT3

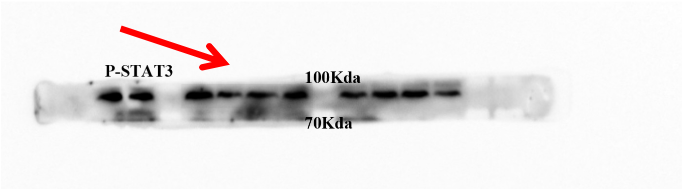


GAPDH

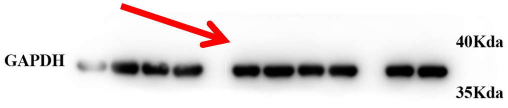

Figure7C

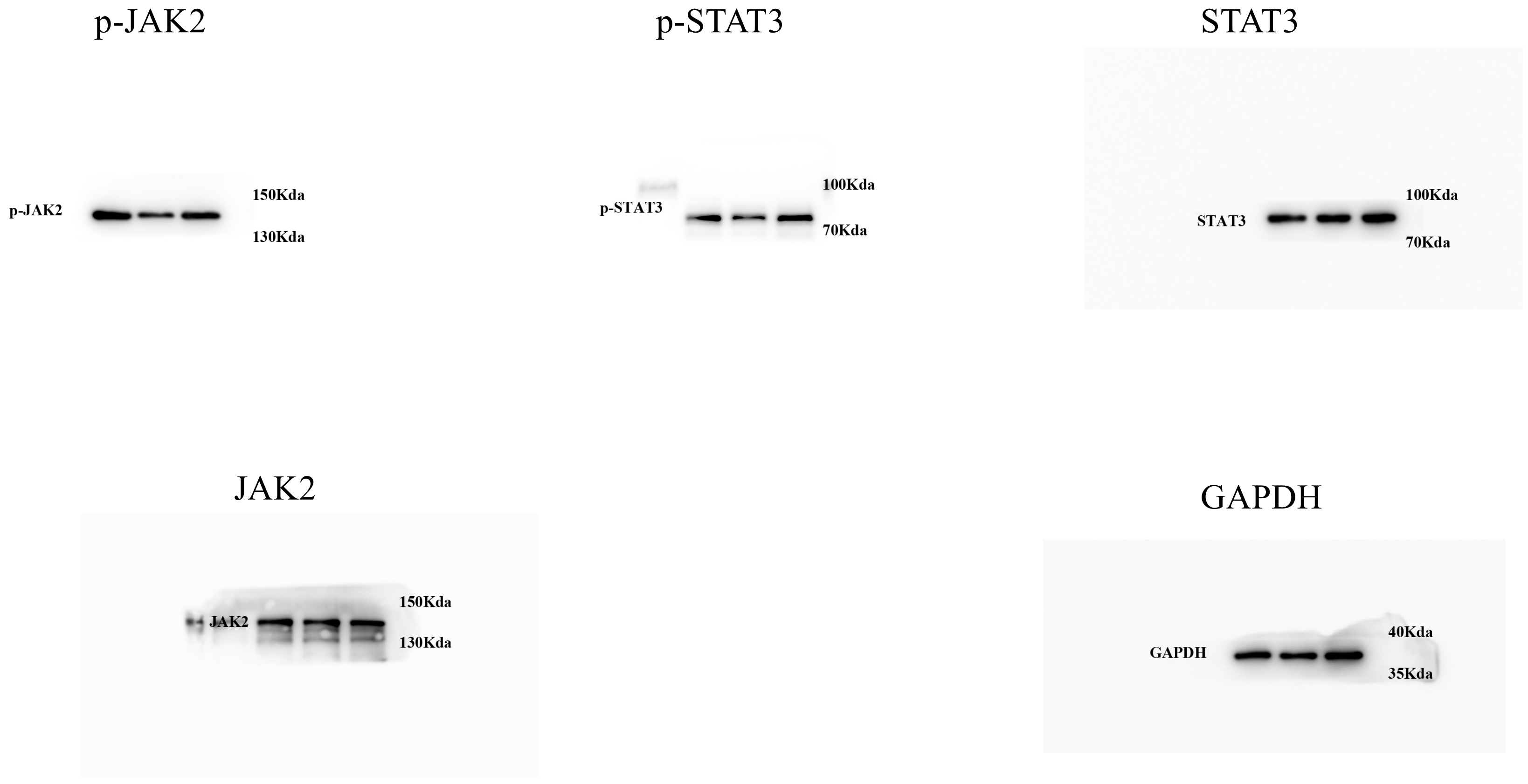
p-JAK2
p-JAK2
150Kda
130Kda
p-STAT3
p-STAT3
100Kda
70Kda
STAT3
STAT3
100Kda
70Kda
JAK2
JAK2
150Kda
130Kda
GAPDH
GAPDH
40Kda
35Kda

Figure7D

p-JAK2

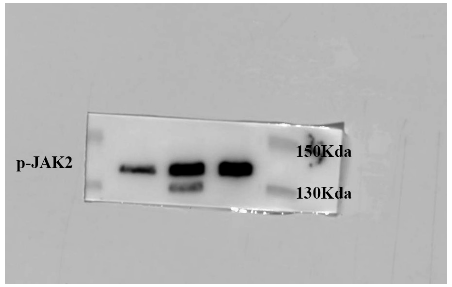


p-STAT3

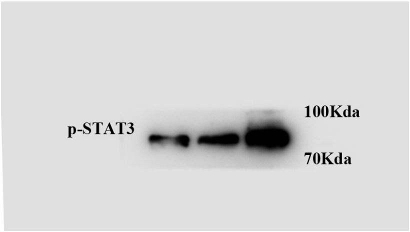


STAT3

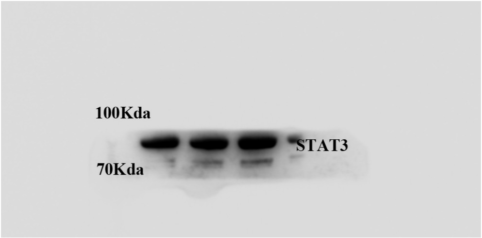


JAK2

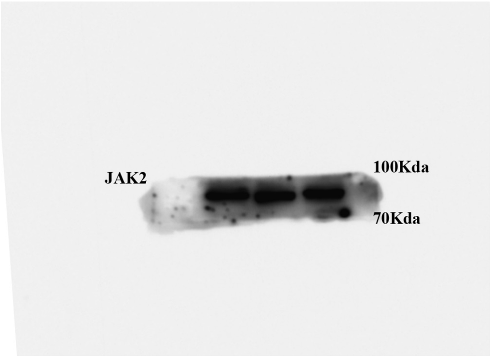


GAPDH

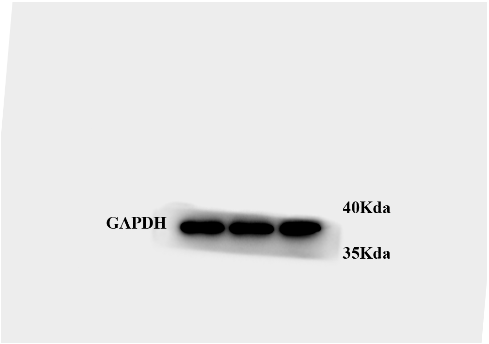

Figure6H

GAPDH CD206 CD163

T24

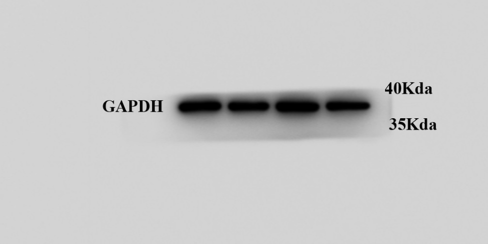


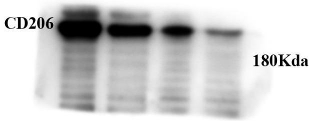


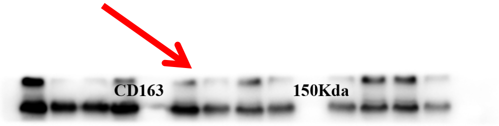

Figure6H

GAPD
H

CD163

CD206

UMUC3

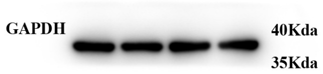


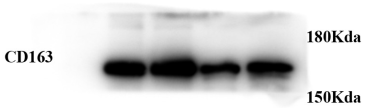


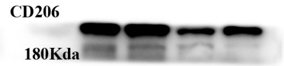

FigureS2A

GAPDH

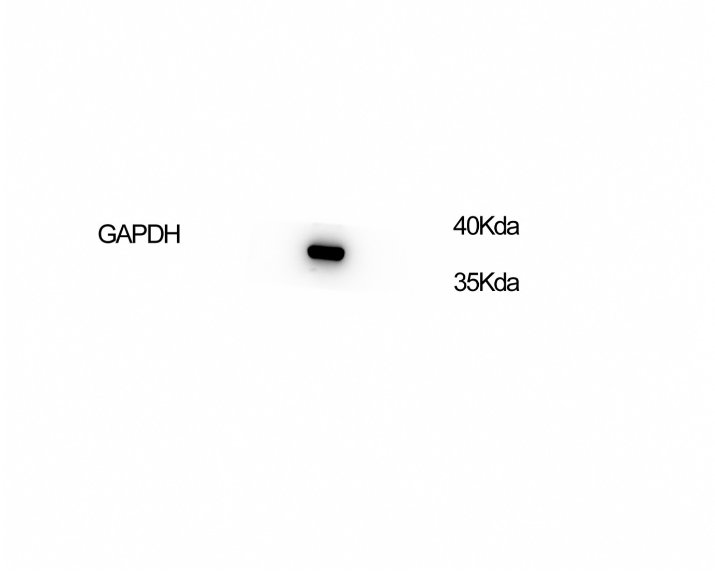


wnt3A

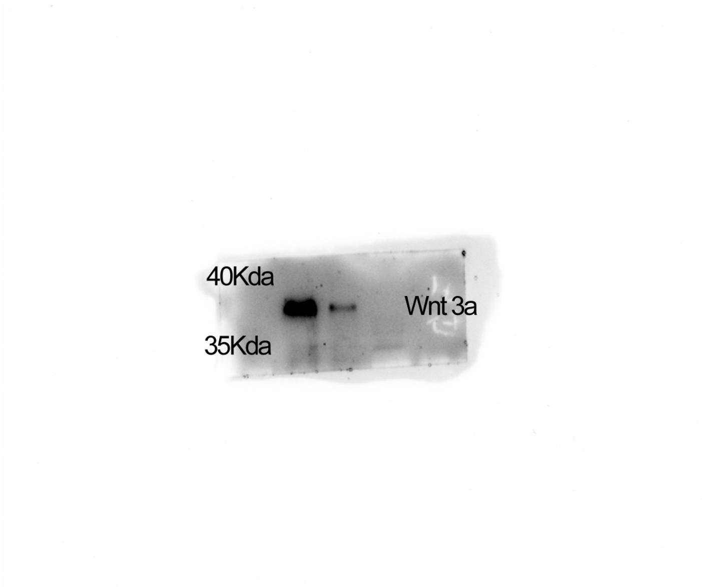

FigureS2B

GAPDH

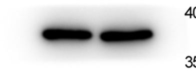


β-catenin

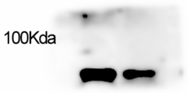

FigureS2C

GAPDH

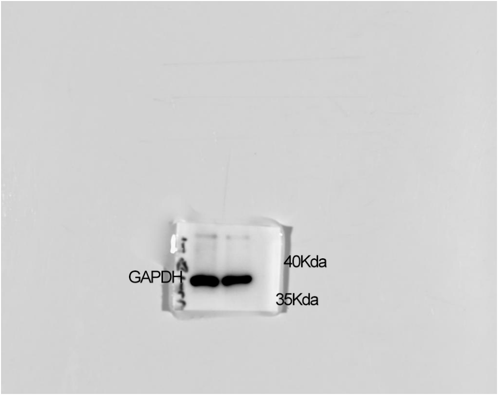


β-catenin

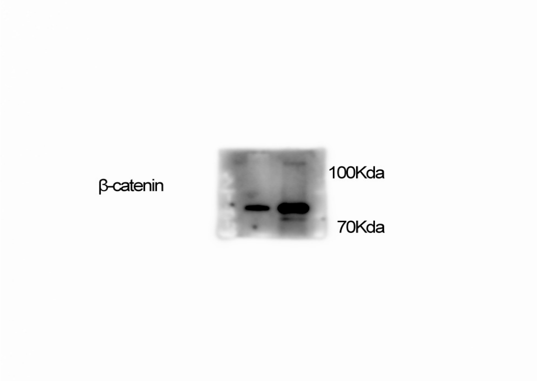

FigureS2D

GAPDH

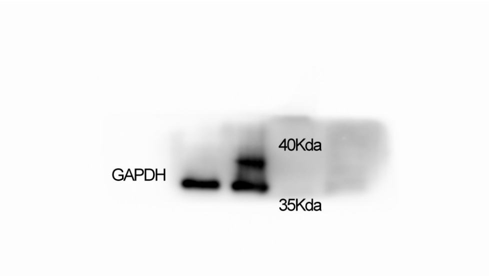


STAT3

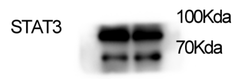


p-STAT3

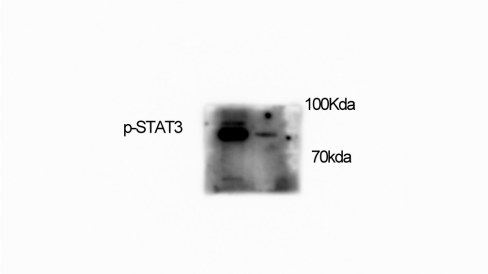


p-STAT6

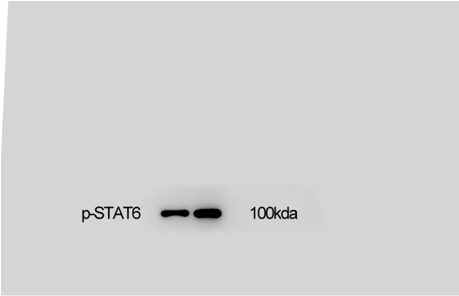


p-STAT1

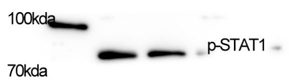


STAT6

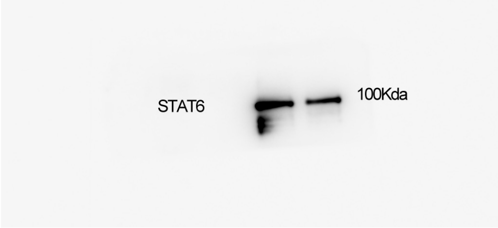


STAT1

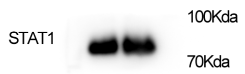

Supplement: Supplementary file 1 [file cancers-15-00131-s001.zip › cancers-2026401-File S1.pdf]
