# Supplementary material for: Sulfatase 2 Affects Polarization of M2 Macrophages through the IL-8/JAK2/STAT3 Pathway in Bladder Cancer
Source: Cancers (Basel). 2022 Dec 26;15(1):131. doi: 10.3390/cancers15010131 (PMC9818157; doi:10.3390/cancers15010131)
Supplement: Supplementary file 1 [file cancers-15-00131-s001.zip › Supplementary Figure S1.pdf]

Supplementary Figure S1

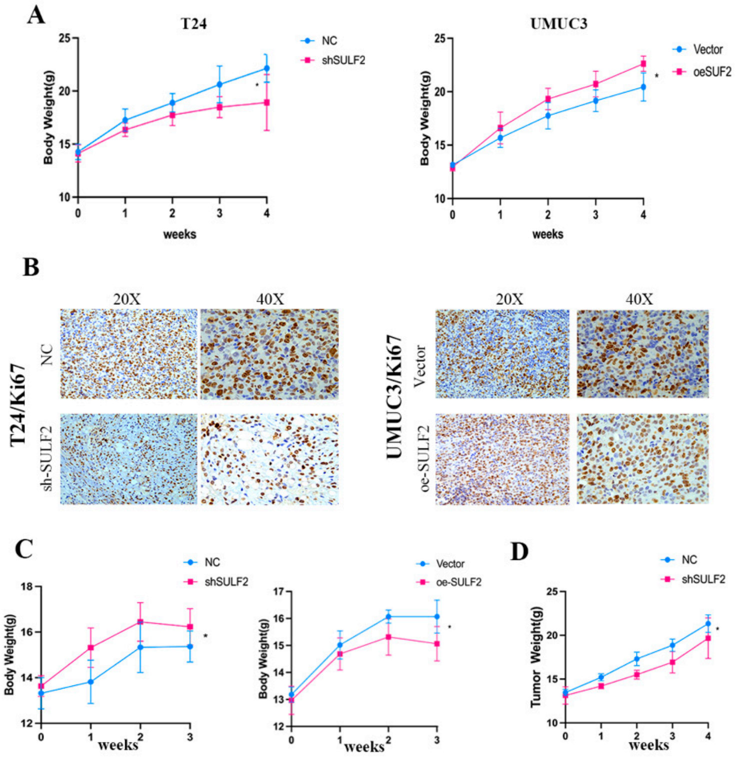

Figure S1 IHC and Body Weight of SULF2 *in vivo* (A) Representative images of Ki67 in tumor cell xenograft after knockdown SULF2 by IHC staining.(B-D) Analysis of tumor growth for the subcutaneous xenotransplant tumor models(b), tail vein lung metastasis model(c) and orthotopic bladder cancer model(d).
