# Supplementary material for: Sulfatase 2 Affects Polarization of M2 Macrophages through the IL-8/JAK2/STAT3 Pathway in Bladder Cancer
Source: Cancers (Basel). 2022 Dec 26;15(1):131. doi: 10.3390/cancers15010131 (PMC9818157; doi:10.3390/cancers15010131)
Supplement: Supplementary file 1 [file cancers-15-00131-s001.zip › Supplementary Figure S2.pdf]

Supplementary Figure S2

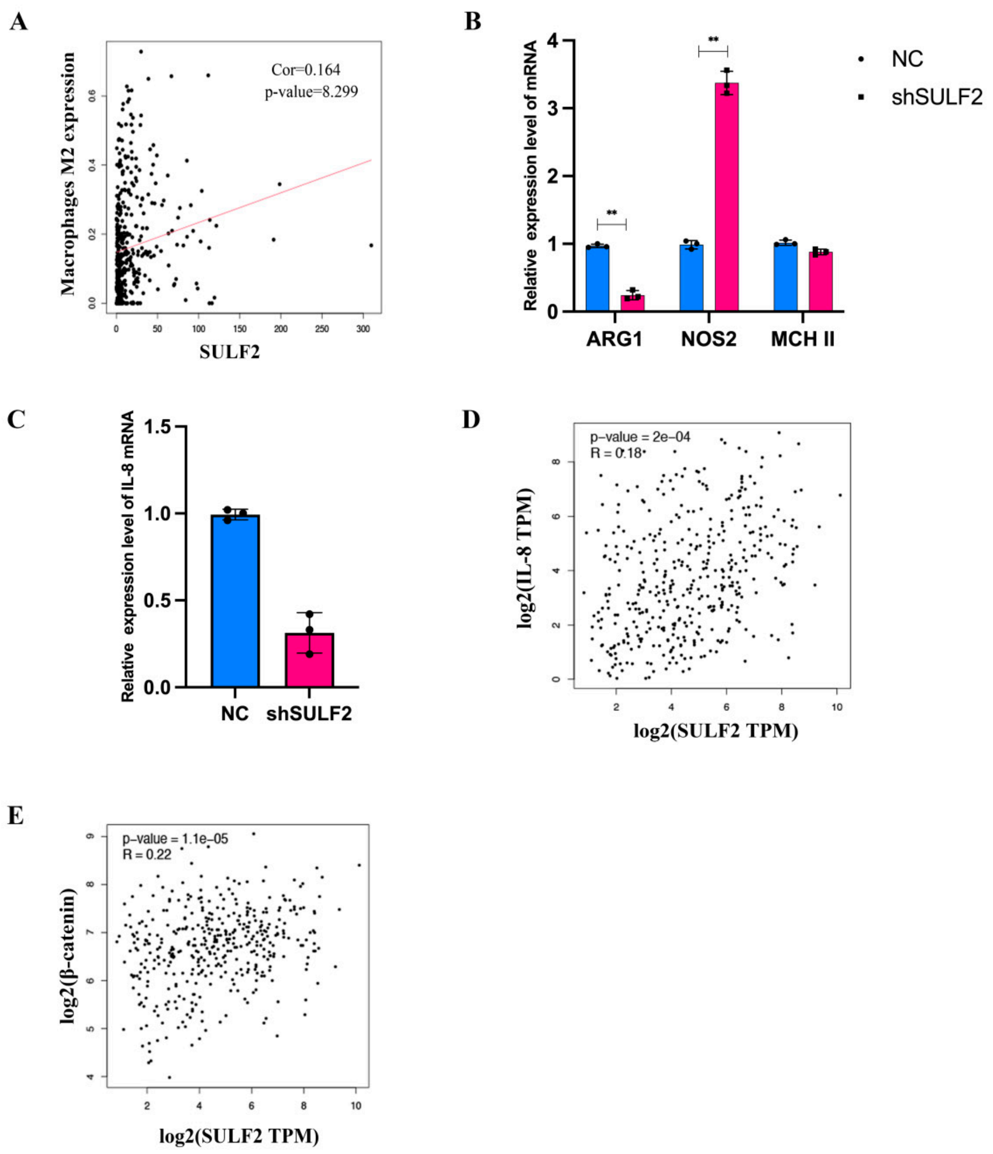


**Figure S2 SULF2 in TCGA database and qPCR validation** (A) SULF2 was positively correlated to M2 macrophages in the TCGA database.(B)Relative mRNA levels of ARG1, NOS2 and MCH II of THP1 in co-culture systems.(C)Relative mRNA levels of IL-8 of MB49 in orthotopic bladder cancer model. (D)The correlation between SULF2 and IL-8 in TCGA database. (D)The correlation between SULF2 and β-catenin TCGA database.
