# Supplementary material for: Sulfatase 2 Affects Polarization of M2 Macrophages through the IL-8/JAK2/STAT3 Pathway in Bladder Cancer
Source: Cancers (Basel). 2022 Dec 26;15(1):131. doi: 10.3390/cancers15010131 (PMC9818157; doi:10.3390/cancers15010131)
Supplement: Supplementary file 1 [file cancers-15-00131-s001.zip › Supplementary Figure S3.pdf]

Supplementary Figure S3

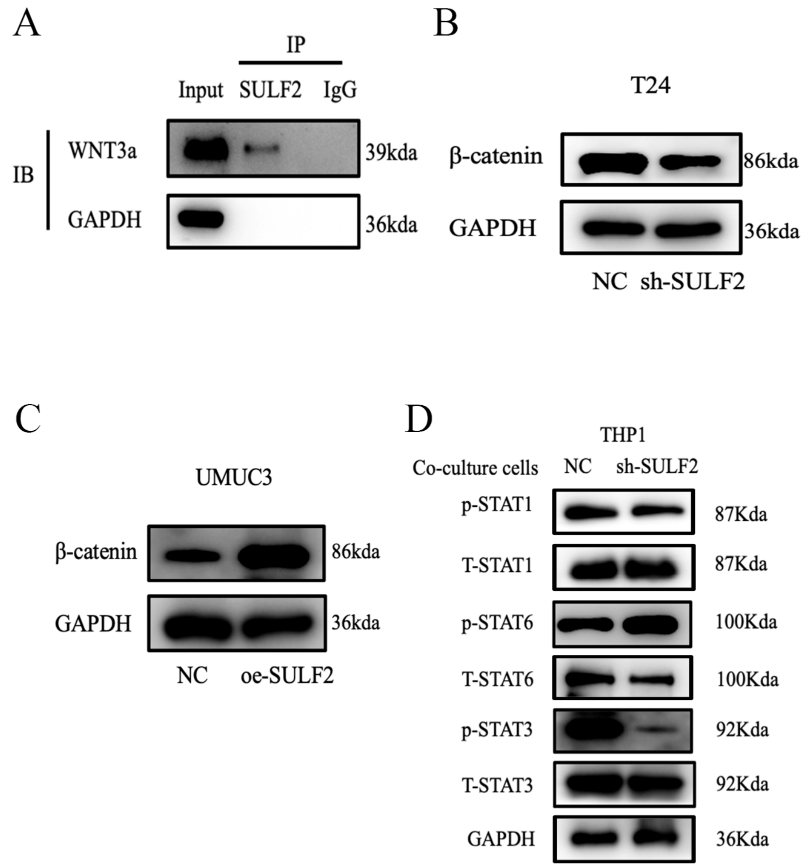

Figure S3 SULF2 regulates the expression of β-catenin (A) IP assay of SULF2 and WNT3A,(B-C) The knockdown and overexpression efficiencies of β-catenin were verified by western blot.(D) The expression levels of STAT family were detected by western blot after knockdown of SULF2
