# Supplementary material for: Sulfatase 2 Affects Polarization of M2 Macrophages through the IL-8/JAK2/STAT3 Pathway in Bladder Cancer
Source: Cancers (Basel). 2022 Dec 26;15(1):131. doi: 10.3390/cancers15010131 (PMC9818157; doi:10.3390/cancers15010131)
Supplement: Supplementary file 1 [file cancers-15-00131-s001.zip › Supplementary Table S1.pdf]

Supplement Table S1: Characteristic of bladder cancer patients in four cohorts.

Characteristic, n (%)	TCGA (n=403)	STPH (n=90)	GSE32894 (n=224)	IMvigor210 (n=348)
Age (years)				
≥65	254 (63.0)	63 (70.0)	154 (68.8)	-
<65	149 (37.0)	27 (30.0)	70 (31.3)	-
Sex				
Male	299 (74.2)	73 (81.1)	163 (72.8)	272
Female	104 (25.8)	17 (18.9)	61 (27.2)	76
Grade				
Low-grade	21 (5.2)	18 (20.0)	129 (57.6)	-
High-grade	378 (93.8)	72 (80.0)	93 (41.5)	-
Unknown	3 (0.7)	-	2 (0.9)	-
Subtype				
NMIBC	3 (0.7)	64 (71.1)	173 (77.2)	-
MIBC	400 (99.3)	26 (28.9)	51 (22.8)	-

STPH: Shanghai tenth people’s hospital; TCGA: The Cancer Genome Atlas Program.
