# Supplementary material for: Sulfatase 2 Affects Polarization of M2 Macrophages through the IL-8/JAK2/STAT3 Pathway in Bladder Cancer
Source: Cancers (Basel). 2022 Dec 26;15(1):131. doi: 10.3390/cancers15010131 (PMC9818157; doi:10.3390/cancers15010131)
Supplement: Supplementary file 1 [file cancers-15-00131-s001.zip › Supplementary TableS3.pdf]

hSULF2 si-1 sense 5'-CCAUGGAGACGAUUUACAATT-3'
hSULF2 si-1 antisense 5'-UUGUAAAUCGUCUCCAUGGAG-3'
hSULF2 si-2 sense 5'-GCUACACAAGAGAGACAAUTT-3'
hSULF2 si-2 antisense 5'-AUUGUCUCUCUUGUGUAGCAG-3'
hSULF2 si-3 sense 5'-GGACCAAGAUGACAAGGAUTT-3'
hSULF2 si-3 antisense 5'-AUCCUUGUCAUCUUGGUCCTC-3'

Primer
SULF2 Forward 5'-AAGGTTGATTCTTTCAGGGAGC-3'
Reverse 5'-AGTGGATCTGAGTTAGGTCATGG-3'
GAPDH Forward 5'- GCCAAGGTCATCCATGACAACTTTGG-3'
Reverse 5'- GCCTGCTTCACCACCTICITGATGIC-3'
ARG1 Forward:5’-TGATGTTGACGGACTGGACC-3′
Reverse 5′-ATCTAATCCTGAGAGTAGCCCTGT-3′
NOS2 Forward:5’-TCCGAGGCAAACAGCACATTC-3′
Reverse 5′-GGGTTGGGGGTGTGGTGATGT-3′
MCH II Forward:5’-ACCTACCCTTCCTCCCTTCTGC -3′
Reverse 5′-ACAACCCCAGGGCACAGACC-3′

mouse-IL-8(CXCL15) Forward:5’-GGCCCAATTACTAACAGGT-3′
Reverse 5′-ATATAGAGGCTTTTCATGCTCA-3′
mouse-GAPDH Forward:5’-TCAACAGCAACTCCCACTCTT-3′
Reverse 5′-CCAGGGTTTCTTACTCCTTGG-3′
